# Fluoroquinolone resistance and mutation profiles in *Mycobacterium tuberculosis* through whole genome sequencing in Fujian province, China

**DOI:** 10.3389/fpubh.2026.1890292

**Published:** 2026-07-09

**Authors:** Yongming Lin, Yong Zhao, Jian Lin, Shufang Lin, Zhisong Dai, Shuzhen Wei

**Affiliations:** 1 Fujian Provincial Center for Disease Control and Prevention, Fuzhou, China; 2 Fujian Provincial Key Laboratory of Zoonosis Research, Fuzhou, China

**Keywords:** drug resistance, fluoroquinolone, gene mutation, *Mycobacterium tuberculosis*, whole-genome sequencing

## Abstract

**Objective:**

This study aimed to characterize fluoroquinolone (FQs) resistance and associated gene mutations in *Mycobacterium tuberculosis* (MTB) isolates from Fujian Province, with the goal of providing evidence to support precise prevention and control strategies for drug-resistant tuberculosis (TB).

**Methods:**

A retrospective analysis was performed on 150 clinical MTB isolates collected from drug resistance surveillance sites. Phenotypic drug susceptibility testing (pDST) for FQs was conducted using the proportion method on solid Löwenstein–Jensen (L–J) media. In parallel, whole-genome sequencing (WGS) was applied to identify resistance-associated gene mutations.

**Results:**

The overall phenotypic resistance rate to FQs was 12.7%, increasing markedly to 26.7% among multidrug-resistant (MDR) strains. The isolates were mainly classified into lineage L2 (60.0%) and L4 (38.7%), with no statistically significant association observed between lineage distribution and drug resistance profiles. Mutations linked to FQs resistance were predominantly identified in the *gyrA* gene (81.8%), primarily occurring at codons 90, 91, and 94. Notably, mutations were also detected in seven phenotypically susceptible isolates.

**Conclusion:**

FQs resistance among MTB isolates in this study is considerable, with a particularly high burden in MDR strains. Resistance is mainly driven by single-point mutations in the *gyrA* gene. Importantly, WGS demonstrates potential for identifying resistance-associated mutations even in phenotypically susceptible isolates, offering valuable insights for early warning of emerging drug resistance and optimization of treatment strategies for MDR/RR-TB.

## 1 Introduction

Tuberculosis (TB) remains one of the most formidable global public health challenges. In 2024, the World Health Organization (WHO) formally classified rifampicin-resistant *Mycobacterium tuberculosis* (MTB) as a “critical priority” pathogen for the first time (1). In the 2025 WHO Global Tuberculosis Report (2), China is categorized as a country with a medium-to-low TB burden. Despite this overall improvement, multidrug-resistant/rifampicin-resistant tuberculosis (MDR/RR-TB) continues to pose a major threat to TB control efforts.

According to WHO treatment guidelines, fluoroquinolones (FQs) are key components of regimens for drug-resistant TB, and are recommended in all-oral short-course therapeutic strategies (3). However, the effectiveness of these regimens depends critically on the accurate detection of FQs resistance, which represents a decisive determinant of treatment outcomes and prognosis in patients with MDR/RR-TB.

Globally, FQs resistance in TB remains at a relatively high level and is disproportionately concentrated among patients with MDR/RR-TB, with evidence suggesting a continuing upward trend (4, 5). In China, FQs resistance is characterized by both a high prevalence and marked regional heterogeneity. Reported resistance rates among MDR-TB strains exceed 34.7%, and in some regions may surpass 50% (6–9). In Fujian Province, spatiotemporal clustering of MDR/RR-TB has been documented (10), and the inappropriate use of FQs during TB treatment remains relatively common (11). Nevertheless, the current epidemiological landscape of FQs resistance in MTB at the provincial level remains insufficiently defined, and its molecular characteristics based on whole-genome sequencing (WGS) have not yet been fully elucidated. Therefore, a comprehensive investigation of FQs resistance patterns and associated gene mutations is urgently needed to support the optimization of treatment strategies for drug-resistant TB.

## 2 Materials and methods

### 2.1 Clinical isolates

**Figure 1:**
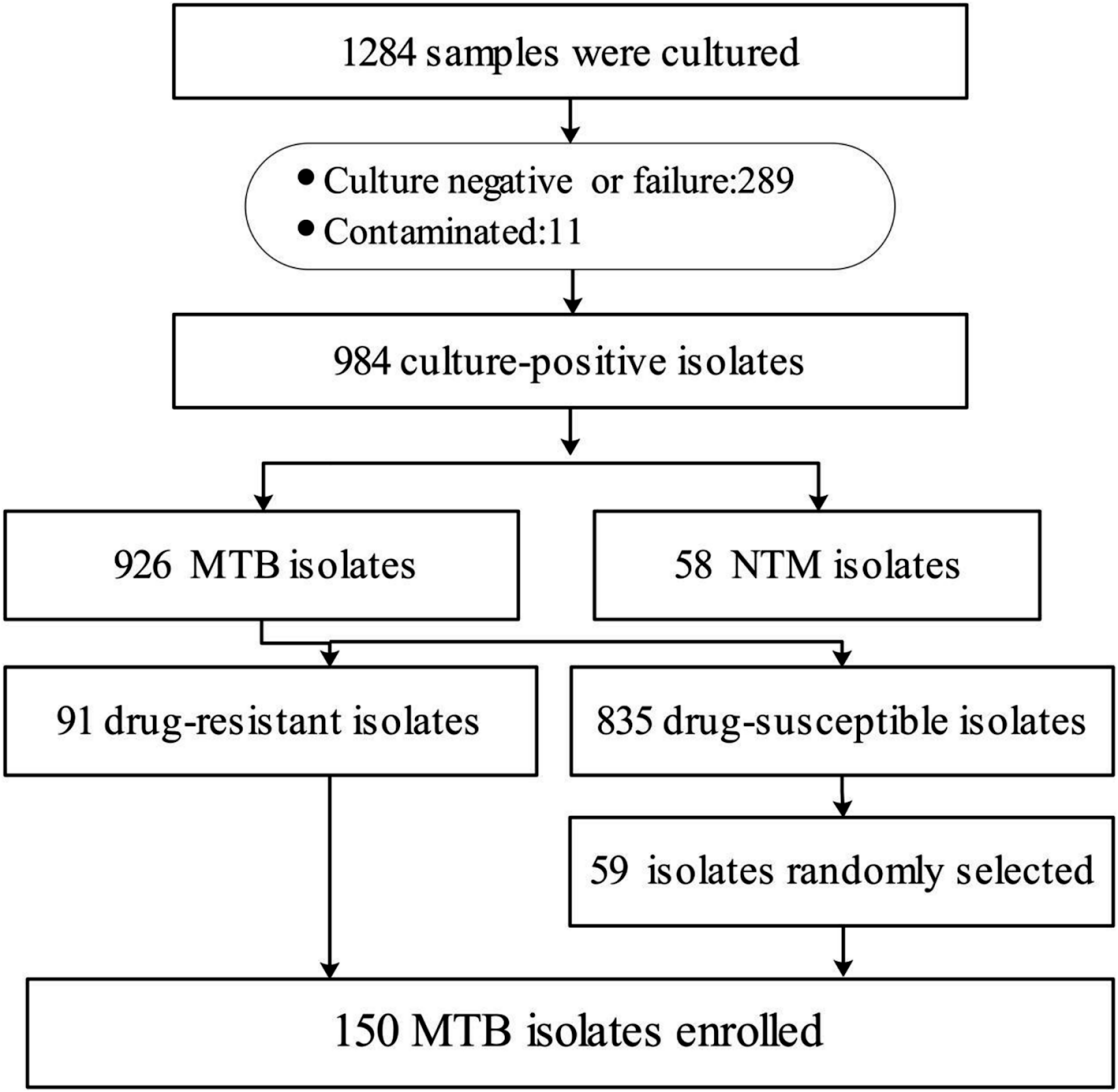
Flow chart of isolates collected in this study. MTB, *Mycobacterium tuberculosis*; NTM, Nontuberculous Mycobacterium.

This study conducted a retrospective analysis of clinical isolates collected from tuberculosis surveillance sites between January 2021 and December 2022. As shown in the flowchart (Figure 1), 91 drug-resistant isolates were successfully obtained, and an additional 59 drug-susceptible strains were randomly selected from 835 phenotypically susceptible isolates. The *Mycobacterium tuberculosis* H37Rv strain (ATCC 27294), provided by the Tuberculosis Reference Laboratory of the Chinese Center for Disease Control and Prevention (China CDC), was used as the reference strain.

### 2.2 Subjects and clinical data

This retrospective study included 150 pulmonary TB patients whose clinical isolates were collected between 2021 and 2022. Baseline demographic characteristics (sex, age, occupation, etc.) and clinical information (including TB treatment history) were obtained using structured questionnaires. Treatment outcome data were retrieved from the electronic database of China’s National Tuberculosis Information Management System. Written informed consent was obtained from all participants prior to data and specimen collection. Of the 150 patients, 116 (77.3%) were male and 34 (22.7%) were female. The age of patients ranged from 18 to 83 years and was categorized as ≤44 years (*n* = 51, 34.0%) and ≥45 years (*n* = 99, 66.0%). A total of 143 patients (95.3%) had no prior history of TB treatment, while 7 (4.7%) had previously received anti-TB therapy. The study protocol was approved by the Ethics Committee of the Fujian Provincial Center for Disease Control and Prevention (FJCDC). All clinical specimens were processed and cultured as described in a previous study (12).

### 2.3 Phenotypic drug susceptibility testing and species identification

Phenotypic drug susceptibility testing (pDST) and species identification were performed as previously described (12, 13). The standard proportion method was used to determine drug susceptibility of *Mycobacterium tuberculosis* (MTB) isolates on Löwenstein–Jensen (L–J) medium, commercially obtained from BASO Biotech (Zhuhai, China). The following anti-tuberculosis drugs were tested at the indicated critical concentrations: isoniazid (INH, 0.2 μg/mL), rifampicin (RIF, 40 μg/mL), ethambutol (EMB, 2 μg/mL), streptomycin (SM, 4 μg/mL), ofloxacin (OFX, 4 μg/mL), kanamycin (KM, 30 μg/mL), capreomycin (CPM, 40 μg/mL), ethionamide (ETO, 40 μg/mL), and para-aminosalicylic acid (PAS, 1 μg/mL). Species identification was performed by growth testing on L-J medium supplemented with 500 μg/mL p-nitrobenzoic acid (PNB) or 5 μg/mL thiophene-2-carboxylic acid hydrazide (TCH). The *M. tuberculosis* H37Rv reference strain was used as a quality control in each experimental batch. All provincial TB reference laboratories in FJCDC and prefecture-level TB laboratories passed annual drug susceptibility proficiency testing organized by the Tuberculosis Reference Laboratory of the China CDC.

### 2.4 Genomic DNA extraction and WGS

Fresh cultures grown on L-J medium were processed using the cetyltrimethylammonium bromide (CTAB) protocol as described in a previous study (12). DNA concentration was measured using a Qubit 2.0 fluorometer (Thermo Fisher Scientific). Qualified DNA libraries were subjected to paired-end sequencing (2 × 150 bp) on an Illumina NovaSeq 6,000 platform, with an average sequencing depth of 200×. For each sample, sequencing output was at least 500 Mb, with Q20 ≥ 95%, Q30 ≥ 90%, GC content ranging from 60% to 65%, and adapter contamination ≤10%.

The WGS pipeline was applied as previously described (14), with the inferred MTB complex ancestor sequence as the reference genome. In brief, the Sickle tool was used for trimming whole-genome sequencing data, and sequencing reads with Phred base quality above 20 and read length longer than 30 were kept for analysis. The retained sequencing reads were mapped to the reference genome with Bowtie2 (v2·4·1), and the SAMtools (v1·6)/VarScan (v2·3·6) suite was used for SNP calling with mapping quality greater than 30. The fixed SNPs (frequency≥75%), excluding those in drug-resistance associated genes and repetitive regions of the genome (e.g., PPE/PE-PGRS family genes, phage sequence, insertion or mobile genetic elements), were used to calculate the pairwise SNP distances. The minimum mutation frequency for heteroresistance in MTB was defined as 10%. Strains with a sequence depth less than 20X or a genome coverage less than 95% were excluded from the analysis.

Mutations associated with resistance to 16 anti-tuberculosis drugs—including INH, RIF, EMB, SM, PZA, amikacin (AM), KM, capreomycin (CM), FQs, ETO, PAS, linezolid, clofazimine, bedaquiline, and delamanid—were analyzed using the online SAM-TB platform^1^ (15). The detected mutations were compared against the mutation catalogue published by WHO (16). Quality-controlled MTB genomic data were further used for phylogenetic analysis and visualization via the same platform. The resulting phylogenetic tree was exported in Newick format and refined using the Interactive Tree of Life (iTOL) platform^2^.

### 2.5 Statistical analysis

Categorical variables were summarized as frequencies and percentages. Comparisons between groups were performed using Pearson’s chi-square test or Fisher’s exact test, as appropriate. A *p*-value < 0.05 was considered statistically significant. All statistical analyses were conducted using SPSS software (version 24.0; IBM Corp., Armonk, NY, USA).

## 3 Results

### 3.1 FQs resistance profiles in pDST and gDST

Among the 150 clinical isolates, 59 (39.3%) were pan-susceptible and 91 (60.7%) were drug-resistant based on phenotypic drug susceptibility testing (pDST). Overall, 19 isolates (12.7%) were resistant to FQs, including 15 multidrug-resistant (MDR) isolates (10.0%; resistant to both rifampicin and isoniazid) and 4 single rifampicin-resistant isolates (2.7%). Among the 15 MDR isolates, 4 (26.7%) showed additional resistance to FQs. Furthermore, 15 FQs-resistant isolates were identified among the remaining 131 rifampicin-susceptible isolates. No statistically significant difference in FQs resistance rates was observed between rifampicin-susceptible and rifampicin-resistant groups (*χ*^2^ = 3.173, *p* = 0.075).

Whole-genome sequencing (WGS)-based drug susceptibility testing (gDST) identified 22 isolates (14.7%) carrying mutations associated with FQs resistance. Among these, mutations were located in *gyrA* (18/22, 81.8%), *gyrB* (3/22, 13.6%), or both genes simultaneously (1/22, 4.5%). Point mutations represented the dominant mutation type, detected in 19 of 22 FQs-resistant isolates (86.3%). These mutations were mainly concentrated at codons 90, 91, and 94 in *gyrA*. In addition, double mutations in *gyrA* were observed in two isolates (9.1%), while one isolate (4.5%) harbored concurrent mutations in both *gyrA* and *gyrB*.

Among the 22 gDST-defined FQs-resistant isolates, heteroresistance was detected in 9 (40.9%), including one isolate that was phenotypically susceptible by pDST. The allele frequencies of resistance-associated mutations in these heteroresistant isolates ranged from 12.7% to 98.3%. Notably, the proportion of isolates harboring FQs resistance-associated mutations was significantly higher among rifampicin-resistant isolates (40.0%, 6/15) than among rifampicin-susceptible strains (11.85%, 16/135) (*χ*^2^ = 5.340, *p* < 0.05).

**Figure 2:**
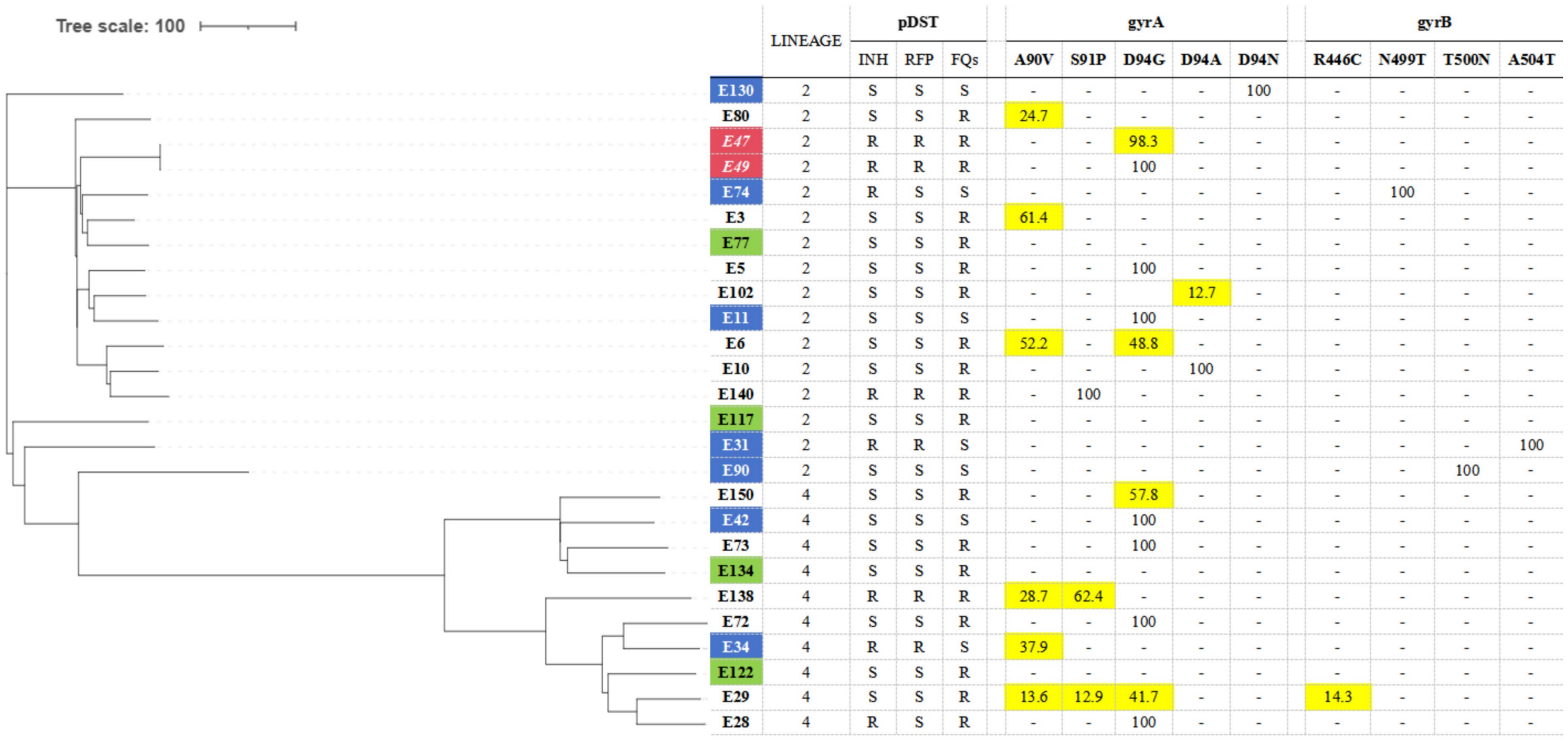
Phylogeny and heteroresistance profiles of 26 FQs-resistant *MTB* isolates by pDST and gDST. (1) Genomically clustered strains (differing by fewer than 12 SNPs) were highlighted in red. (2) MTB isolates with phenotypic susceptibility but genotypic resistance to FQs were shown in blue, while those exhibiting phenotypic resistance despite genotypic susceptibility were indicated in green. (3) Allele frequencies of FQs resistance-conferring mutations in *gyrA* and *gyrB* were displayed on the right. Heteroresistance were highlighted in yellow.

Overall, 26 isolates were classified as FQs-resistant when integrating both pDST and gDST results. Among them, 22 carried resistance-associated mutations, whereas the remaining 4 exhibited phenotypic resistance without identifiable mutations. The phylogenetic distribution and allele frequency patterns of FQs resistance-associated mutations are shown in Figure 2.

### 3.2 Transmission of FQs-resistant isolates

To assess recent transmission, phylogenetic analysis was performed on 22 genotypically FQs-resistant isolates using a ≤ 12 single nucleotide polymorphism (SNP) threshold. A single transmission cluster comprising two FQs-resistant isolates (E49 and E47) was identified (Figure 2). The resistance in the cluster isolates may be driven by the *gyrA* D94A mutation, with mutant allele frequencies of 100 and 98.3%, respectively. Accordingly, genomic data indicated that recent transmission accounted for 4.5% (1/22) of FQs-resistant TB cases.

### 3.3 Association between lineages with MDR and FQs resistance mutations

Lineage analysis based on WGS showed that most isolates belonged to lineage 2 (L2; 90/150, 60.0%) and lineage 4 (L4; 58/150, 38.7%), while lineage 1 (L1) and lineage 3 (L3) were each represented by a single isolate (0.7% each).

Among the 15 MDR isolates identified by pDST, 10 belonged to L2 (11.1% within L2) and 5 to L4 (8.6% within L4), with no statistically significant difference in lineage distribution (*χ*^2^ = 0.240, *p* = 0.624). Similarly, among the 22 FQs-resistant isolates identified by gDST, 14 were L2 (15.6% of L2) and 8 were L4 (13.8% of L4), with no significant difference in mutation distribution between L2 and L4 lineages (*χ*^2^ = 0.087, *p* = 0.769).

### 3.4 Association between gene mutation and pDST

Concordance between gDST and pDST was observed in 139 of 150 isolates, yielding an overall agreement rate of 92.7%. Among these, 15 isolates were classified as resistant and 124 as susceptible by both methods. The remaining 11 isolates (7.3%) showed discordant results, including 4 isolates with phenotypic resistance but no detectable genotypic mutations and 7 isolates with phenotypic susceptibility but carrying resistance-associated mutations.

Using pDST as the reference standard for FQs resistance detection, WGS-based gDST demonstrated a sensitivity of 78.9%, specificity of 94.7%, positive predictive value (PPV) of 68.2%, and negative predictive value (NPV) of 96.9%. Overall agreement between the two methods was substantial (kappa = 0.69; *χ*^2^ = 71.820, *p* < 0.01) (Table 1).

**Table 1:** Association between mutations and FQs-resistance in MTB isolates.

Gene mutation type			Number of isolates	pDST for FQs	
			*N* (%)	Resistant *N* (%)	Susceptible *N* (%)
Confidence level of mutation-resistance association (16)					
*gyrA* Gene	Single SNP	*gyrA*_D94G	9 (6.0)	7 (36.8)	2 (1.5)
		*gyrA*_A90V	4 (2.7)	3 (15.8)	1 (0.8)
		*gyrA*_D94A	2 (1.3)	2 (10.5)	0
		*gyrA*_D94N	1 (0.7)	0	1 (0.8)
		gyrA_S91P	1 (0.7)	1 (5.3)	0
	Double SNP	*gyrA*_A90V+	1 (0.7)	1 (5.3)	0
		*gyrA*_D94G			
*gyrB* Gene	Single SNP	*gyrB*_N499T	1 (0.7)	0	1 (0.8)
*gyrA*+*gyrB* Gene	Multiple Combined SNPs	*gyrA*_A90V+	1 (0.7)	1 (5.3)	0
		*gyrA*_S91P+			
		*gyrA*_D94G			
Confidence level of mutation-resistance association (16) not found					
*gyrB* Gene		*gyrB*_T500N	1 (0.7)	0	1 (0.8)
		*gyrB*_A504T	1 (0.7)	0	1 (0.8)
		*gyrB*_R446C	1 (0.7)	1 (5.3)	0
No mutation			128 (85.3)	4 (21.1)	124 (94.7)
Total			150 (100.0)	19 (100.0)	131 (100.0)

Importantly, among the 131 isolates classified as FQs-susceptible by pDST, WGS detected resistance-associated mutations in 7 isolates (5.3%). These included six mutation patterns: *gyrA* A90V (*n* = 1), D94N (*n* = 1), D94G (*n* = 1), and *gyrB* mutations (*n* = 3).

## 4 Discussion

FQs are cornerstone agents in the treatment of multidrug-resistant/rifampicin-resistant tuberculosis (MDR/RR-TB). However, the emergence of FQs resistance critically compromises the feasibility of short-course regimens (e.g., BPaL-M), necessitating longer, more toxic, and less effective alternative therapies. This shift substantially increases the risk of progression to pre-extensively drug-resistant (pre-XDR) or extensively drug-resistant (XDR) TB, thereby posing significant therapeutic challenges and adversely affecting clinical outcomes.

As broad-spectrum antimicrobial agents, FQs are extensively prescribed for respiratory and other infections, particularly in empirical treatment of community-acquired pneumonia. This widespread use exerts strong selective pressure, thereby accelerating the emergence of FQs-resistant MTB strains (17). Consequently, a proportion of TB patients may acquire FQs resistance prior to formal diagnosis and treatment initiation. In addition, environmental drivers, including extensive use of FQs in animal husbandry and food production, may contribute to environmental contamination and facilitate the selection and dissemination of resistant strains (18). Moreover, regional variation in circulating MTB genetic backgrounds further shapes resistance dynamics and mutation patterns, collectively contributing to the marked geographic heterogeneity observed in FQs resistance (19–21).

FQs resistance has become a major global challenge in TB control. A meta-analysis covering 22 countries reported a pooled prevalence of 27% (22%–33%) among MDR-TB patients, with particularly high levels in the Western Pacific (35%) and Southeast Asia (32%) regions (22). Moreover, resistance continues to show an increasing trend worldwide (23, 24). National surveillance data in China have shown that resistance to levofloxacin and moxifloxacin among rifampicin-resistant isolates reaches 26.1 and 21.9%, respectively (6).

In the present study, we evaluated the prevalence and transmission characteristics of FQs resistance among MTB isolates obtained through routine drug resistance surveillance. Based on gDST, 14.7% of isolates carried FQs resistance-associated mutations, among which 40.9% exhibited heteroresistance. Transmission accounted for 4.5% (1/22) of FQs-resistant TB cases, indicating that most resistance likely arises through independent acquisition rather than recent transmission.

The overall phenotypic FQs resistance rate (12.7%) observed in this study was higher than that reported in Shanghai (7.4%) (17) and Zhejiang (4.6%) (25). The FQs resistance rate among MDR isolates (26.7%) was comparable to the national surveillance data (26.1%, 2013–2020) (6), the historical baseline in the same region (26.9%, 2010) (26), and data from Beijing (27.7%) (7). However, it remained lower than reports from several high-burden settings, including Iran (39.7%) (27), India (33%) (28), Chongqing (41%) (29), Jiangxi (50%) (30), and Zhengzhou (55.1%) (31), while slightly higher than that reported in low-burden settings such as the United Kingdom (23.9%) (5). Overall, these findings suggest that the selective pressure driving FQs resistance in Fujian Province is broadly consistent with the national average.

In response to this growing threat, the WHO has designated MTB as a priority pathogen and emphasized the urgent need to strengthen surveillance and control of FQs resistance (1, 2). Continued expansion of resistance undermines TB control strategies, particularly for MDR-TB management. Therefore, rational use of FQs in clinical practice, along with strict avoidance of inappropriate empirical prescriptions, is essential to reduce selection pressure (3, 16). Furthermore, continuous monitoring of FQs efficacy in MDR/RR-TB treatment regimens and implementation of targeted surveillance strategies remain critical public health priorities.

WGS-based lineage analysis showed that the dominant MTB lineages in this study were L2 (Beijing genotype, 60.0%) and L4 (Euro-American genotype, 38.7%), consistent with the general epidemiological pattern in China. Although Beijing genotype strains have historically been associated with increased virulence and drug resistance potential (32–35), our results showed no significant association between lineage and MDR status (*p* > 0.05). Similarly, no significant difference in FQs resistance rates was observed between L2 and L4, consistent with recent WGS-based studies suggesting that FQs resistance is not strongly lineage-dependent (6, 8, 25).

At the molecular level, mutations were predominantly located in the *gyrA* gene (81.8%), particularly at codons 90, 91, and 94, consistent with previous reports (26, 27, 29, 31). Among these, Asp94Gly and Asp94Asn are well-established markers of high-level FQs resistance (16). Although *gyrB* mutations accounted for a smaller proportion (13.6%), their clinical significance remains less clearly defined, warranting further investigation. Accordingly, *gyrA* should remain the primary target for molecular surveillance of FQs resistance.

The mutation profile observed in this study is highly consistent with previous findings in Fujian Province (26) and other regions (6, 7, 29, 31). Notably, one isolate carried concurrent mutations in both *gyrA* and *gyrB*, which may indicate higher-level resistance or complex resistance evolution, requiring closer clinical attention (19).

Overall, we observed strong concordance between WGS-based predictions and pDST results for FQs resistance, consistent with previous studies (4, 20, 33, 34). However, discrepancies remain. Consistent with literature (36) reports on sequencing sensitivity, the use of Illumina whole-genome sequencing or use of GridION platform may reduce the overall accuracy for detecting any drug-resistant MTB. Although WGS is the focus of this study, targeted next-generation sequencing approaches are becoming increasingly relevant for clinical implementation (37). Further evaluation of these platforms for mycobacterial identification and drug resistance detection is warranted.

In four phenotypically resistant isolates, no known resistance-conferring mutations were detected. This may be explained by several factors: (1) incomplete coverage of the WHO mutation catalogue (2nd edition), which may not include rare or novel variants, particularly outside*gyrA*/*gyr*B or in genes such as mfpA and efpA (6, 16, 19); (2) efflux pump-mediated resistance mechanisms contributing to low-level resistance (38); and (3) heteroresistance, where mixed populations fall below WGS detection thresholds (21, 33).

Conversely, six distinct mutation patterns were identified in seven phenotypically susceptible isolates, a phenomenon increasingly reported in recent studies (5, 17, 25, 28). These discordant isolates may represent an intermediate evolutionary state, where mutations increase MIC values without exceeding clinical breakpoints (16, 19), or may reflect mutations with weak or uncertain phenotypic impact (e.g., *gyrA* A90V). Therefore, interpretation of WGS-based predictions should be performed cautiously, particularly for variants of uncertain significance, and ideally complemented with MIC testing or functional validation (16, 21).

Heteroresistance poses challenges across three key areas. In terms of treatment decisions, clinicians must remain aware of the limitations of conventional drug susceptibility testing and prudently design combination regimens that cover potentially resistant subpopulations. In WGS interpretation, laboratory staff need to recognize the sensitivity limits of genotypic assays and integrate genotypic findings with phenotypic results. For transmission surveillance, epidemiologists require more refined genomic analyses to distinguish true transmission from the confounding effects of heteroresistance.

From a public health perspective, such cryptic resistance poses a potential risk. Under FQs exposure, these strains may rapidly acquire full resistance, leading to treatment failure and further transmission of drug-resistant TB (8, 28). This risk is particularly relevant in regions with high baseline FQs resistance burden (10, 13), highlighting the importance of early molecular detection as a potential warning system.

Several limitations should be acknowledged. First, the relatively small sample size may limit the generalizability of our findings to the broader provincial epidemiological context, and sampling limitations may have led to an underestimation of clustering. Second, this study lacked longitudinal follow-up and clinical outcome data. Such data would be valuable for understanding whether isolates harboring resistance-associated mutations but classified as phenotypically susceptible eventually progress to overt resistance or are associated with treatment failure. Furthermore, the absence of epidemiological linkage data limits our ability to explore temporal dynamics and causal associations. Third, the observed discordance (7.3%) between gDST and pDST was not further validated by MIC testing or targeted sequencing, which could have clarified the underlying resistance mechanisms.

## 5 Conclusion

Overall, our findings demonstrate a substantial burden of MDR-TB and FQs resistance in this study. The rational use of FQs is essential to mitigate further resistance emergence. No significant association was observed between MTB lineage and either MDR or FQs resistance. Mutations in *gyrA* represent the principal mechanism of FQs resistance. The application of WGS offers significant potential for early detection of resistance evolution, supporting precision-guided management and individualized treatment strategies for drug-resistant TB.

## Data Availability

The datasets presented in this study can be found in online repositories. The names of the repository/repositories and accession number(s) can be found at: https://ngdc.cncb.ac.cn/gsa/search?searchTerm=CRA029438.

## Footnotes

1
https://samtb.uni-medica.com/index

2
https://itol.embl.de/
